# Recovery in personality disorders: the development and preliminary testing of a novel natural language processing model to identify recovery in mental health electronic records

**DOI:** 10.3389/fdgth.2025.1544781

**Published:** 2025-04-03

**Authors:** Giouliana Kadra-Scalzo, Jaya Chaturvedi, Oliver Dale, Richard D. Hayes, Lifang Li, Shaza Mahmood, Jonathan Monk-Cunliffe, Angus Roberts, Paul Moran

**Affiliations:** ^1^ Institute of Psychiatry, Psychology and Neuroscience, King’s College London, London, United Kingdom; ^2^ Sussex Partnership NHS Foundation Trust, Worthing, United Kingdom; ^3^ Centre for Academic Mental Health, Population Health Sciences Department, Bristol Medical School, University of Bristol, Bristol, United Kingdom

**Keywords:** personality disorder, recovery, electronic health records, work, mental health, natural language processing

## Abstract

**Introduction:**

The concept of recovery is of great importance in mental health as it emphasizes improvements in quality of life and functioning alongside the traditional focus on symptomatic remission. Yet, investigating non-symptomatic recovery in the field of personality disorders has been particularly challenging due to complexities in capturing the occurrence of recovery. Electronic health records (EHRs) provide a robust platform from which episodes of recovery can be detected. However, much of the relevant information may be embedded in free-text clinical notes, requiring the development of appropriate tools to extract these data.

**Methods:**

Using data from one of Europe's largest electronic health records databases [the Clinical Records Interactive Search (CRIS)], we developed and evaluated natural language processing (NLP) models for the identification of occupational and activities of daily living (ADL) recovery among individuals diagnosed with personality disorder.

**Results:**

The models on ADL performed better (precision: 0.80; 95% CI: 0.73–0.84) than those on occupational recovery (precision: 0.62; 95%CI: 0.52–0.72). However, the models performed less acceptably in correctly identifying all those who recovered, generally missing at least 50% of the population of those who had recovered.

**Conclusion:**

It is feasible to develop NLP models for the identification of recovery domains for individuals with a diagnosis of personality disorder. Future research needs to improve the efficiency of pre-processing strategies to handle long clinical documents.

## Introduction

Personality disorders are common mental health disorders, with a community prevalence of ∼7% (1). They are associated with significant distress, impairment in functioning, increased psychiatric and physical comorbidity, and reduced life expectancy (2–6). Remission in individuals with personality disorder is most commonly defined as the decrease or absence of clinical symptoms. Symptomatic remission among people with a diagnosis of personality disorder is possible but usually occurs over a number of years (7). Yet, within the field of mental health, remission of symptoms is only one aspect of change, and it may not capture other areas of importance to individuals as they recover (8–10). Research has highlighted the enduring nature of impairment in functional, relational, and work domains for people diagnosed with personality disorder (11, 12). Indeed, changes in these domains are important and can be linked to improvement in the quality of service users' lives (12). Framing disorders in terms of recovery also helps to reduce the stigma that mental disorders are untreatable. However, to date, there is sparse research that has examined non-symptomatic recovery among services users with a personality disorder diagnosis. Previous research has been primarily qualitative in nature (12) and has relied on small, non-random samples (7, 13, 14), limiting the generalizability of the findings. From an epidemiological perspective, investigating non-symptomatic recovery in the field of personality disorders has been challenging due to the requirement of reliable case detection and the complexity of capturing the occurrence of recovery over time. Electronic health records (EHRs) potentially provide a robust platform from which the occurrence of episodes of recovery can be detected over long periods of time for a defined population cohort. However, much of the routinely recorded information on recovery is contained in free-text clinical notes, rather than in the structured (such as drop-down menus) portions of EHRs, making it difficult to extract and analyze such information. Therefore, we set out to develop and evaluate natural language processing (NLP) models for the identification of recovery among individuals diagnosed with personality disorder, using free-text data from de-identified EHRs contained in Clinical Records Interactive Search (CRIS)—one of Europe's largest electronic health records databases (15).

## Materials and methods

### Data source

To develop the models, we used data from the CRIS system, which was developed in 2008 to allow researchers to search and retrieve anonymized South London and Maudsley NHS Foundation Trust (SLAM) EHRs, encompassing four ethnically diverse London boroughs, Lambeth, Southwark, Lewisham, and Croydon, a population of approximately 1.36 million (14, 15), with approximately 500,000 cases currently represented in the system. CRIS contains a large volume of diverse and longitudinal (with EHRs used across all SLaM services since 2006) data, from both structured fields and free-text (such as progress notes) and has been successfully used to study service users with personality disorder and their outcomes in previous work (16, 17). CRIS operates within a strict governance framework designed and implemented with service user involvement and is approved as a database for secondary analysis by the Oxford C Research Ethics Committee (18/SC/0372) (15, 18). This project received input from inception to write-up from the SLAM Service User and Carers Advisory Group (19).

### Cohort

We identified all services users who had received a diagnosis of personality disorder (ICD 10: F60.x) between 2007 and 2022.

### Procedures

One approach to extract information about recovery would be to use a lexicon of terms that potentially indicate improvement or deterioration, combined with span categorization, a rules-based or machine learning approach to categorize spans of text containing these words as actually indicating improvement or deterioration. Spans, in this context, refer to segments or sections of text, which contain a starting and an endpoint. For example, in the sentence, “he has been unemployed for 5 years”, spans could be various sections of the sentence, such as “been unemployed”, “5 years”, or even “unemployed for 5 years”. Spans can have varying lengths but are generally shorter sections of a bigger sentence. Spans of text that indicate recovery can be very varied in their language, referring to a wide variety of social and cultural situations to indicate improvement or deterioration. This makes it challenging to create a comprehensive lexicon. Our approach therefore did not use a fixed lexicon of terms, aiming for more flexibility in identifying varied spans of text about recovery. However, the spans of text about recovery did not have defined borders, as might be seen in complete sentences. Therefore, this design decision gave us greater flexibility when identifying spans, although imprecision in how a span length was defined made training a model harder. We refer to the task of identifying, labeling, and coding spans as annotating spans and the labeled, coded, spans as annotations.

**Figure 1:**
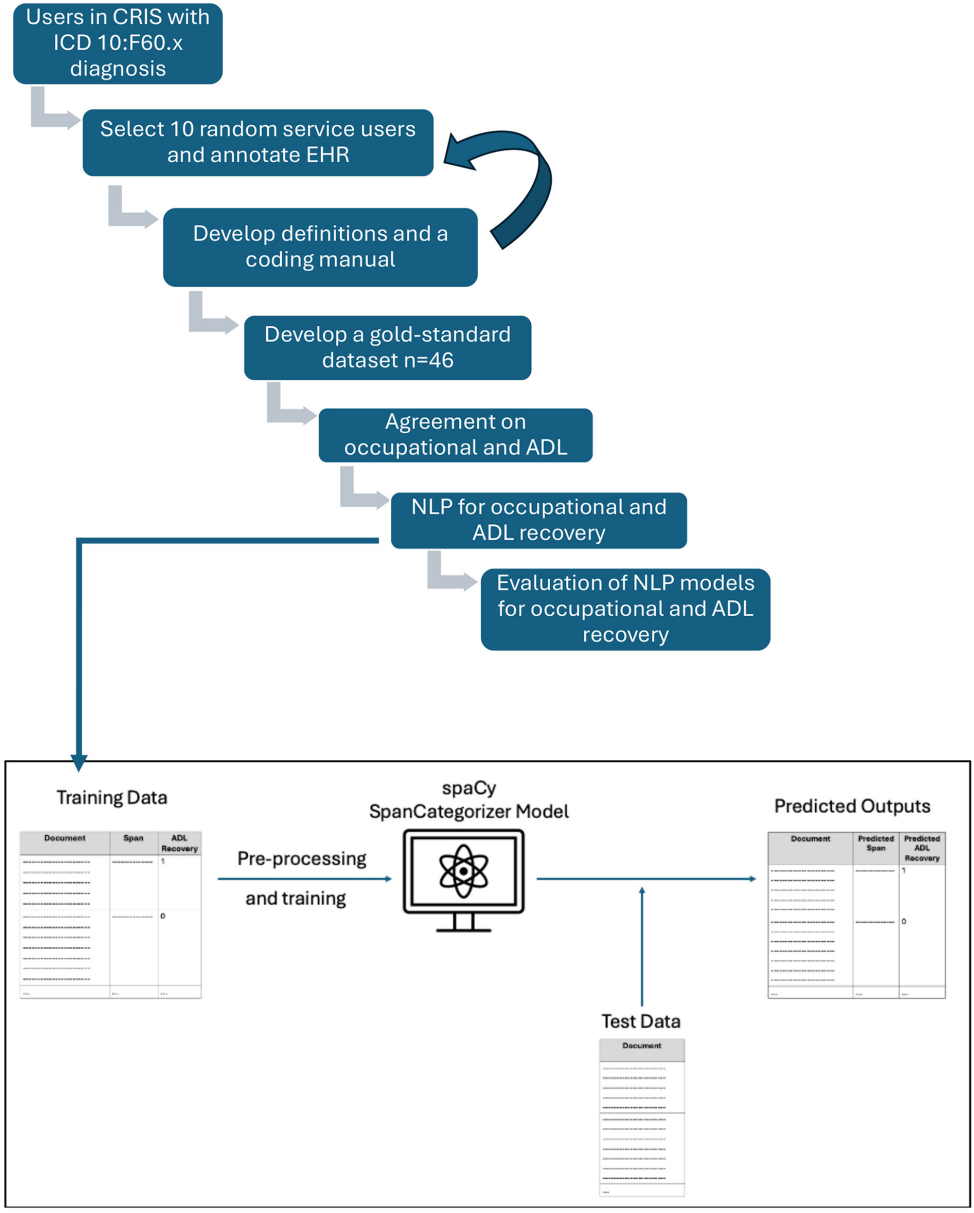
Process of gold-standard generation and NLP development.

To develop the NLP models, the work underwent two distinct phases. Figure 1 summarises the entire process from both phases. In summary, during Phase 1, we sought to establish whether information on recovery was available in the EHRs and to create a coding framework and a manual, which would enable human coders to identify recovery and distinguish between different types of recovery, reflecting the nuanced nature of service users' experiences. This framework was designed to allow for consistent coding of EHRs and was used to generate a manually coded, gold-standard dataset. During Phase 2 we explored whether this data could be used to develop NLP models to reliably ascertain this information over the entire EHR database.

#### Phase 1

Due to the novel nature of this project, we began Phase 1 by investigating whether information on recovery was available in the clinical notes of the EHRs by manually reading anonymized records for ten randomly selected people with a personality disorder diagnosis. Spans of text with relevant information were extracted by human coders and coded for the relevant domain as per five categories which are outlined below. This was an iterative process and instances where ambiguous annotations were identified were resolved by discussion with the multidisciplinary team.

We investigated five domains of recovery—the definitions were derived using existing literature and clinical expertise from our multidisciplinary team involving two senior psychiatrists with expertise in the treatment of personality disorder (PM and OD) and two researchers (GK-S and RH) with extensive experience in using EHRs to examine service users from this specific population. Supplementary Material 1 summarizes the definitions of the subdomains of recovery and their corresponding coding rules.

1. Non-specified recovery is referred to recovery indicated in the notes without an explicit reference to specific domains. Information in the text would be around the person's ability to attend to any aspect of everyday functioning.
2. Social recovery is referred to finding evidence in the text to indicate the presence of at least one meaningful social relationship (intimate partner, family member, friend).
3. Occupational recovery is referred to evidence in the text of work, volunteering, vocational training, and study, inclusive of hobbies and caring commitments, which are consistent and meaningful.
4. Activities of daily living (ADL) recovery is referred to the ability to organize and manage aspects of daily life such as dressing, hygiene, transportation, shopping, finances (bills, managing assets), meal prep, home maintenance, communication with others (phone, email), and medications.
5. Personal recovery is referred to evidence in the text around the person's grounding and their relationship to self such as increased self-awareness and confidence.

Once recovery definitions and a coding manual were finalized, we built a manually coded, gold-standard dataset, which was required to build the NLP models for the identification of recovery. The dataset contained clinical information on a number of randomly selected patients, manually coding their entire clinical history from first to last clinical contact available through their clinical EHR. The inter-annotator agreement was estimated by an independent clinician (JM-C) not involved in the development of the coding on a subset, using % agreement and Cohen's kappa.

#### Phase 2

In Phase 2 of the study, the NLP models were developed to ascertain spans of text mentioning recovery. Automating this process from a manual to a computerized approach has the advantage of being able to code very large numbers of documents rapidly, facilitating research on large datasets. The manually coded gold-standard data was randomly split into two subsets, at the patient level: a training set (80%) and a held-back test set (20%).

A common task in NLP is the extraction of spans of texts from documents. This involves identifying spans of text in a document and classifying them according to predefined categories. One variation of this, named entity recognition (NER), focuses on short spans of text, such as person or organization names that are generally represented by small numbers of tokens. When considering recovery, we are interested in larger spans of text, such as phrases or whole sentences. Span categorization is generally a two-step process. Step 1 involves the identification of the spans of interest, in this case, spans about recovery. Step 2 is the classification of these identified spans into predefined classes, such as the types of recovery.

**Figure 2:**
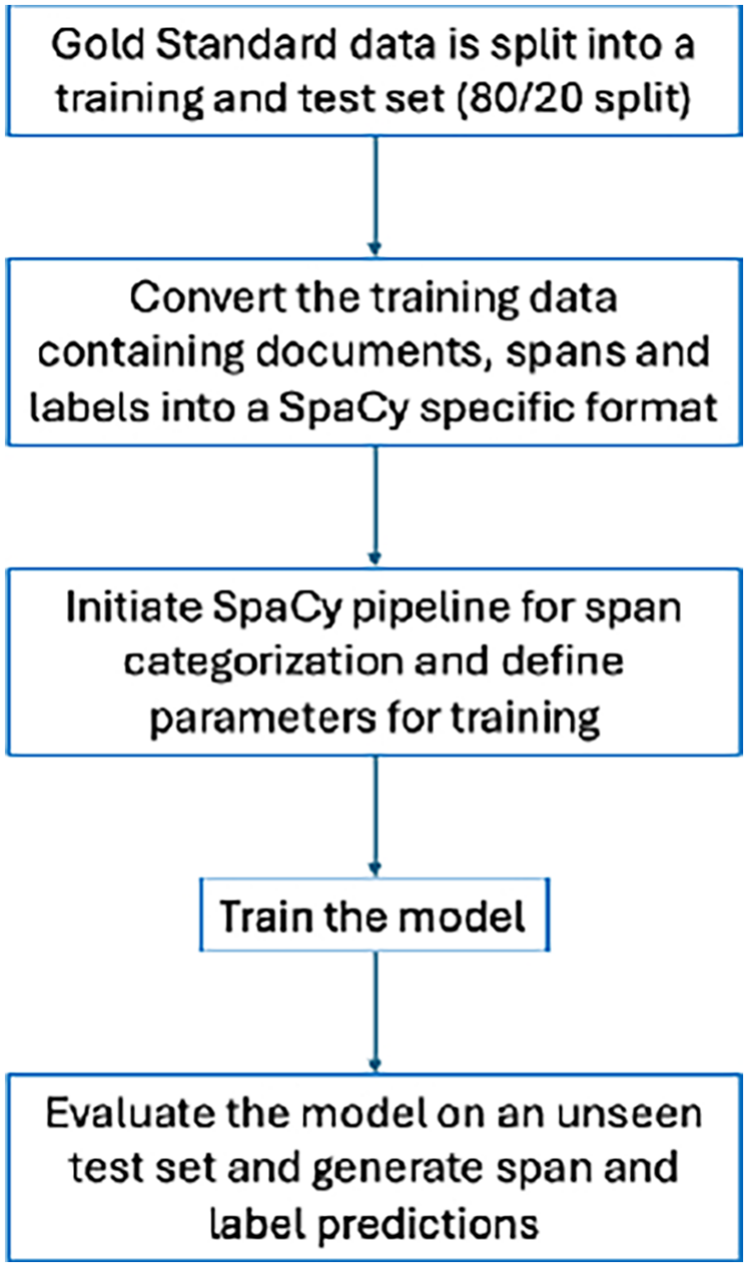
Process of training and evaluating NLP models.

Our span categorization method involved two steps: (1) identifying spans of text in a document and (2) classifying them according to predefined categories. For spans, we used phrases or whole sentences. For span categorization, we used SpaCy's (https://spacy.io/) SpanCategorizer (https://spacy.io/api/spancategorizer) component. The SpanCategorizer was trained on the training set, with the primary objective of identifying and classifying spans of text containing information related to recovery. The training enabled the model to learn both the relevant spans about recovery, as well as their corresponding classes. Upon completion of training, the SpanCategorizer model was able to identify potential spans of text about recovery in the previously unseen test set and suggest appropriate classes for these spans. Figure 2 summarizes our approach.

## Results

### Data extraction and annotation

Using structured and free-text data, we identified 11,164 service users in CRIS with a diagnosis of personality disorders (ICD-10: F60.x) between 2007 and 2022. We manually coded the entire EHRs of 46 randomly selected service users with a personality disorder diagnosis, ranging from patients with four documents in their records to other patients with over 3,600 documents in their EHRs, with an average of 120 documents per patient. In total, approximately 300 h were spent on manual annotation to produce the dataset. Information on recovery (relating to patient functioning) for this specific service user group did exist in the EHRs. Table 1 outlines the number of text spans coded in each recovery domain as indicating the presence or absence of recovery (as of 26/09/22) for all 46 patients. There was a considerable variation in the information available in the EHRs for each of the five domains. More specifically, the number of annotations available for the domains of non-specified and personal recovery was too low to allow for NLP development. In addition, we noticed a great variation in the language used to discuss social recovery, which meant that this domain may need special attention when developing an NLP model. Therefore, for the purpose of training an NLP model to identify and extract recovery, we chose to focus on two (occupational and ADL recovery) of the five domains, which contained sufficient annotations to allow us to train the models. The inter-annotator agreement for the two domains was high: 95.5% (95% CI: 0.91–0.96) (Cohen's kappa 91%, 95% CI: 0.82–0.92) for ADL (358 spans), and 93.6% (95% CI: 0.89–0.96)(Cohen's kappa 82%, 95% CI: 0.72–0.90) occupational recovery (205 spans).

**Table 1:** The number of text spans coded in each domain of recovery and in total for 46 patients with a personality disorder diagnosis.

Domain	Text spans indicating that recovery is present	Text spans indicating that recovery is absent	Total
Non-specified	36	30	66
Social	5,876	982	6,858
Occupational	1,647	403	2,050
ADL	2,000	1,580	3,580
Personal	379	196	575
Total	9,938	3,191	13,129

### Data description

The documents at their full length (maximum length of 31,665 words) proved challenging for the training of the model, resulting in no predictions being generated. This could be due to longer documents introducing more noise and variability, as well as the potentially larger number of potential spans, which can be computationally intense and challenging to identify relevant spans. To address this issue, limiting the text length was considered, with the assumption that shorter text might allow for more focused training of the model, thus reducing the number of potential spans for the model to predict. For this reason, two text length variations were explored. The first approach included only 200 characters on either side of the labeled text span about recovery, resulting in a mean length of 414 words (min, 4; max, 1,270). The second approach attempted to split documents into sentences, using only the sentence containing the text span about recovery. However, this method was unsuccessful at accurate sentence segmentation, due to inconsistent use of punctuation and capitalization of the first word of the sentences, which is what most NLP approaches rely on to split documents into sentences. This resulted in sentences with a mean length of 917 words (min, 3; max, 6,464). Models trained using the second approach did not generate any predictions, while the first approach (200 characters on either side of the span) did.

In addition to document length, the length of the annotated recovery spans was also considered. These spans had a mean length of 87 words (min, 10; max, 870), leading to a similar challenge as mentioned previously, where the model did not generate any predictions. To overcome this, a variation using shorter spans (<150 words, with a mean length of 72) was used. This 150-word limit, approximately equivalent to the average length of two sentences, was deemed sufficient for capturing the relevant information about recovery. Notably, 88% of the annotated spans fell within this 150-word limit.

### Performance metrics

Six variations of the model were trained using the shorter document length and annotated spans. These variations were chosen to investigate and compare the model's performance in distinguishing between different types of recovery (occupational vs. ADL) and to assess whether focusing on a single span type (“recovery present”) would improve performance. Two categories of performance metrics are reported, corresponding to the two stages of span categorization: span identification and span classification. For both categories of performance metrics, the following scores have been used: Precision measures how often the model was correct when it identified recovery-related spans of text. For example, if the model identified 100 spans as relevant, and 80 of those were actually relevant, the precision would be 80%. Recall measures what proportion of all the relevant spans the model successfully found. For instance, if there were 100 relevant spans in the documents, and the model found 70 of them, the recall would be 70%. The F1 score combines precision and recall into a single number that helps understand the overall performance. It balances how good the model is at both finding relevant spans (recall) and avoiding incorrect identifications (precision). A perfect F1 score of 1.0 would mean the model found all relevant spans without making any errors.

## Performance on span identification

Lenient metrics (i.e., the gold-standard spans do not have to exactly match the predicted spans, a partial match will also be considered a match) have been employed here, as most instances of inaccuracy involved only minor discrepancies, such as missing a word or two in the identified spans, and slight variations in span boundaries are potentially inconsequential to the identification of recovery-related information. It also allows for consideration of overlapping spans, so a prediction is considered correct if there is overlap with the gold standard, rather than requiring exact boundary matches. Table 2 describes the different models and their performance in predicting spans. Macro averages (the arithmetic mean of each class's performance metrics) were used so that equal importance is given to both classes, when two classes exist (Models 1–3).

**Table 2:** The models, their descriptions, and performance metrics (macro average) on span prediction, including 95% confidence intervals.

Model	Description	F1 score (95% CI)	Precision (95% CI)	Recall (95% CI)
Model 1	Identify and extract spans related to occupational recovery	0.17 (0.11–0.23)	0.52 (0.37–0.66)	0.10 (0.06–0.14)
Model 2	Focus on activities of daily living (ADL) recovery	0.40 (0.34–0.44)	0.74 (0.68–0.80)	0.27 (0.23–0.30)
Model 3	Identify spans related to either occupational or ADL recovery	0.24 (0.21–0.26)	0.69 (0.64–0.74)	0.15 (0.12–0.16)
Model 4	Extract only “recovery present” spans for occupational recovery	0.26 (0.20–0.32)	0.62 (0.52–0.72)	0.17 (0.12–0.20)
Model 5	Extract only “recovery present” spans for ADL recovery	0.58 (0.52–0.63)	0.80 (0.73–0.84)	0.45 (0.40–0.50)
Model 6	Extract “recovery present” spans for either occupational or ADL recovery combined	0.30 (0.26–0.32)	0.56 (0.52–0.60)	0.20 (0.18–0.22)

## Performance on span classification

Each predicted span was classified into labels/classes denoting the class they belong to. A description of the labels has been included in Table 3. The performance metrics for these models on this span label classification are detailed below. The metrics reported are macro averages, which ensure equal weighting to both classes in the models that are distinguishing between two classes.

**Table 3:** Performance metrics on span classification for the different models.

Model	Class description	F1 score (95% CI)	Precision (95% CI)	Recall (95% CI)
Model 1	Spans related to occupational recovery are classified as “recovery present” or “recovery absent” (two classes)	0.17 (0.10–0.23)	0.60 (0.44–0.75)	0.10 (0.06–0.13)
Model 2	Spans related to ADL recovery are classified as “recovery present” or “recovery absent” (two classes)	0.43 (0.38–0.48)	0.87 (0.82–0.91)	0.29 (0.25–0.32)
Model 3	Spans related to either occupational or ADL recovery are classified as “recovery present” or “recovery absent” (two classes)	0.28 (0.25–0.30)	0.84 (0.80–0.88)	0.16 (0.14–0.18)
Model 4	Spans related to occupational recovery are labeled as “recovery present” only (one class)	0.39 (0.32–0.44)	1 (1–1)	0.24 (0.19–0.28)
Model 5	Spans related to ADL recovery are labeled as “recovery present” only (one class)	0.68 (0.63–0.71)	1 (1–1)	0.51 (0.46–0.55)
Model 6	Spans related to occupational or ADL recovery are labeled as “recovery present” only (one class)	0.47 (0.44–0.49)	1 (1–1)	0.31 (0.28–0.32)

It is important to note here that Models 1–3 classify the spans into two classes, denoting presence and absence of recovery. Models 4–6, on the other hand, are only predicting one class, i.e., the presence of recovery. For this reason, the latter models show a precision of 1, since the models are only predicting a single class, which is the only class present in the training data. Consequently, when these models make a prediction, it is always correct, leading to a precision of 1. However, these models display a low recall, which indicates that the models are failing to identify many instances where they should have applied this label. However, it is important to note that for Models 4–6, the main focus was on the span prediction task rather than span classification, so the former holds more weight when evaluating the models' performance. It is also worth noting that a label is predicted, regardless of whether these spans match exactly.

## Discussion

This is the first study that has aimed to ascertain functional recovery in mental health service users with a personality disorder diagnosis using EHRs. In line with previous literature looking at rehabilitation language in mental health records (20), we found that useful, codable, information on recovery for this specific service user group not only exists, but we were also able to ascertain information pertinent to subdomains of recovery such as social, occupational, activities of daily living (ADL) and personal recovery. However, we observed substantial variation in the language used, and this had a considerable impact on the process of extracting data from the EHRs. Therefore, following a consultation process with the service user group, we decided to focus on two specific recovery domains—occupational and ADL.

With effective psychological treatment, symptoms of personality disorder, such as impulsive behavior and self-harm, can improve relatively quickly (21). However, it can take many years before people achieve satisfactory stability in occupational activity and relationships (22). Yet recovery in these domains is very important to individuals and should inform care planning. Occupational recovery is essential because activity provides individuals with a sense of purpose, identity, self-efficacy, structure, and of course, income (23). Moreover, employment enables economic independence, reducing the stress and stigma that may otherwise be exacerbated by financial dependency. Thus, the ability of clinicians to routinely detect and record the occurrence of these indicators of recovery is very important for charting a service user's progress through treatment.

Having trialed several different methods to identify information on occupation and ADL recovery in the electronic records, we ultimately used the shorter document lengths and annotated spans, to train several models for domain identification and classification. Overall, the models were better at distinguishing whether recovery was present (focusing on a single span) than distinguishing between different types of recovery (present vs. absent). In addition, although the recall performance of all models was low to moderate, the models on ADL performed better than those on occupational recovery. There are two potential explanations for this. Firstly, we were reliant on the language used by clinicians in the free-text records. Traditionally in clinical practice, ADL is an important clinical domain in judging symptomatic remission and functional recovery, therefore the frequency with which ADL language was used in the clinical records was greater than language relating to occupational recovery. Therefore, compared to the number of text spans for occupational recovery, we were able to generate a greater number of text spans for ADL which could be used to train the models. Secondly, we detected a greater variation in the language used to describe occupational recovery, and this may also have impacted the model training.

The models performed two tasks: firstly identifying relevant text spans discussing recovery (Table 2) and then classifying these spans as indicating either the presence or absence of recovery (Table 3). The first task, i.e., identification of relevant spans proved to be the more challenging aspect, with the model failing to generate predictions for approximately 50% of the population. Where spans were successfully identified, the subsequent classification demonstrated acceptable precision, with 60%–87% of cases correctly labeled as recovered or not recovered. However, the recall metrics in Table 3 should be interpreted in the context of Table 2's performance, as the classification task was dependent on successful span identification. Therefore, the lower recall values reflected both classification errors and more significantly, the challenge of span identification, which may be attributed to factors such as data complexity and document length. While these results demonstrate the feasibility of automated recovery identification from clinical text, they also highlight the need for improvements, particularly in the initial span identification task, beginning with improved data collection and preprocessing strategies.

### Limitations

Although coding the documents for 46 patients allowed us to capture a substantial amount of information, it is possible that for some of the less frequently mentioned types of recovery, coding a larger number of clinical records would have optimized the NLP model development. Coding the documents Several important limitations need to be considered. A key and lengthy component of the methodology involved limiting the text lengths considered for span identification. While the original documents remained unchanged, we decided to look at specific shorter sections of the documents, limited to 200 characters on either side of the span about recovery. However, it is unclear whether such an approach could be feasibly implemented in the future, without prior knowledge of the location of relevant spans. Alternative approaches were considered, such as excluding the longest 50% of documents, however, this did not sufficiently reduce document length and would have resulted in eliminating more documents which would have compromised the size of data available for training. A potential solution for this could involve segmenting long documents into chunks of 400–500 characters. The model could then be run on each of these chunks to identify spans about recovery, and the efficacy of this chunking method and its impact on model performance could then be examined. Furthermore, the models also identified a small portion of spans within the test data that were not included within the gold standard but were still indicative of recovery, such as “getting along well with friends,” “has been doing well,” and “had a good day at work.” In addition, the models captured a large proportion of false negatives, and although span classification displayed reasonable precision in most models, low recall may have occurred because of the absence of spans. This could indicate that a principled definition of spans along linguistic lines, for example, verb and noun phrases, could make it easier for any automated span detection to identify the relevant parts of the text before categorizing.

### Implications for future research

Electronic health record data may, in principle, act as a rich source of data for the detection of such indicators and our work has shown that it is feasible to develop NLP models for the identification of selected domains of recovery among individuals diagnosed with personality disorder. Between 60% and 87% of people identified by the NLP app as having recovered had indeed recovered. However, the models were less than acceptable in correctly identifying all those who recovered—generally missing at least 50% of the population of those who had recovered. Further work is clearly needed to refine the models, to ensure their recall improves. From a research perspective, the ability to analyze a large volume of documents and potentially identify instances of recovery could enhance our understanding of recovery patterns among large patient cohorts and reveal new insights into factors affecting patient recovery. From a bioinformatics perspective, the task of span classification was heavily impacted by the task of span identification, and the performance of the models suggests that document length significantly impacts span identification. Future work should, therefore, consider preprocessing strategies to handle long clinical documents, such as text chunking methods where the document is split into predetermined chunks before being processed by the model. Additionally, while utilizing state-of-the-art large language models such as GPT can be a promising alternative, this was not feasible during this study as privacy constraints within the hospital's secure computing environment required data to remain within the system, therefore limiting both the available models and computational power.

## Data Availability

The data are not publicly available due to the information governance framework and REC approval in place concerning CRIS data use. Requests to access the datasets should be directed to Dr. Giouliana Kadra-Scalzo, giouliana.kadra@kcl.ac.uk.
