# Supplementary material for: Recovery in personality disorders: the development and preliminary testing of a novel natural language processing model to identify recovery in mental health electronic records
Source: Front Digit Health. 2025 Apr 3;7:1544781. doi: 10.3389/fdgth.2025.1544781 (PMC12003297; doi:10.3389/fdgth.2025.1544781)
Supplement: Supplementary file 1 [file Table1.pdf]

Supplementary material 1- Summary of the definitions of the subdomains of recovery and their corresponding coding rules

Domain	Areas covered	Coding rules
**Recovery**	Recovery indicated in the notes without explicit reference to specific domains	<u>Code if (either 1 or 0):</u><br>– Events are related to functional, rather than symptomatic, change<br><u>Code 1:</u><br>– Ability to attend to any aspect of everyday functioning<br>– Increased ability to attend to any aspect of everyday functioning<br><u>Code 0:</u><br>– Difficulty is indicated in relation to any aspect of everyday functioning<br>– Decline in any aspect of everyday functioning
**Social domain**	At least one meaningful social relationship (intimate partner, family member, friend)	<u>Do not code if:</u><br>– Description of generalised difficulties in social functioning are not relevant (unless presence/absence of meaningful relationship is indicated)<br>– Relationships are professional<br>– If a family member or friend is mentioned in the text without evidence to suggest contact between them and the patient (this includes if they are giving collateral information or expressing concern regarding the patient to professionals), unless they are a current spouse/partner<br>– Any reference to an anonymised individual (QQQQQ) should not be interpreted as a social relationship (family member, friend), unless their identity is explicit in the snippet or somewhere in the document<br><u>Code 1:</u><br>– Presence of social relationship is indicated (regardless of any

		relationship difficulties present)<br>Code 0:<br>– Absence or loss of social relationship is indicated
**Occupational domain**	Evidence of work, volunteering, vocational training, or study. Inclusive of hobbies and caring commitments. Consistent, meaningful	Code if (either 1 or 0):<br>– Patient is being treated in forensic mental health services (i.e. low, medium, high secure units), regardless of formal/incentivised structure<br>Do not code if:<br>– Description of generalised difficulties in occupational functioning (unless evident that patient engaged in occupation, where if difficulties are present then still code as 1)<br>Code 1:<br>– Evidence of commitment to activity, such as active (present) engagement, enrolment/acceptance, start date etc.<br>– Meaningful hobbies and caring commitments included<br>Code 0:<br>– Evidence suggesting lack of occupational activity<br>– Loss of any occupational activity<br>– Patient expresses a desire to commit to/engage in an occupational activity
**ADL domain**	Ability to organise and manage aspects of daily life such as dressing; hygiene; transportation; shopping; finances (bills, manage assets); meal prep; home maintenance; communication with others (phone, email); medications	Code if (either 1 or 0):<br>– Levels of functioning in ADLs are indicated due to current circumstances (e.g. financial circumstances, homeless vs settled, result of physical or cognitive disability)<br>– Patient is engaging in ADL activities/assessments on the ward<br>Code 1:

Supplementary material 1- Summary of the definitions of the subdomains of recovery and their corresponding coding rules

		– Evidence for ADL (not targeted/planned)<br>– Patient described as kempt, appropriately dressed etc included<br>– Patient is supported to attend to ADL<br><u>Code 0:</u><br>– Patient is struggling to meet any ADL
**Personal domain**	Insight into self, relationship to self	<u>Code if (either 1 or 0):</u><br>– Evidence is distinct from symptomatic change<br>– Reference to insight into mental health problems/diagnosis/personality traits; self-criticism/self-esteem; or sense of self/self-concept<br><u>Code 1:</u><br>– Some or increasing insight into self or difficulties<br>– Some or increasing self-esteem or sense of self/identity<br><u>Code 0:</u><br>– Limited insight into self or difficulties<br>– Indication of poor self-esteem or sense of self/identity
